# Recherches Anatomico-Pathologiques sur la Médécine Pratique, &c.

**Published:** 1825-04-01

**Authors:** 


*Recherches Anatomico-pathologiques sur la Medecine Pratique, &c.* Par C. F. TACHERON, M.D.

[Third Article, continued from Vol. IV. p. 761.]

---

"Observatio id filum ad quod dirigi debent Medicorum ratiocinia.—*Baglivi.*

---

I. PERICARDITIS. In the fourth volume of our Analytical Series, we brought down the analysis of M. Tacheron's work, to the section on "PERICARDITIS," to which subject we now invite the attention of our readers.

The causes of this formidable inflammation, are the same as of other internal phlogoses—but we believe it is more frequently connected with a rheumatic diathesis, or metastasis of rheumatism than with any other cause. The depressing passions, anxieties of mind, and over-exertions of the intellectual faculties are no unfrequent determining causes of this disease, especially in its chronic and more insidious form. Like other inflammations, it is acute, subacute, or chronic in its march—much more frequently in the two latter than in the first form. It is also very frequently complicated with pleurisy, peripneumony, and even gastritis. Its symptoms, termination, and pathology, will be best gathered from a faithful narrative of cases, with their dissections.

*Case* 1. Madame de ———, 35 years of age, of robust constitution, and formerly in good health, was confined, in an easy and natural manner, on the 13th of June, 1819. About this time, a phlegmonous tumour, which had been growing some time, suppurated under the right arm. The lochia ceased rather suddenly, without any inconvenience, but the patient remained weak for some days after delivery. At length, her strength was fast recovering, the wound healing, and every thing announced complete recovery, when one day she found herself excessively thirsty, and drank a large quantity of water. This was on the 22nd of June. She immediately experienced a great sense of oppression, and one hour afterwards she presented the following phenomena;—viz. extreme præcordial anxiety—inability to lie down for a moment—lips discoloured at intervals—face slightly swelled—copious warm sweats, especially on the face—extreme difficulty of breathing—inability to spit out—pulse small, concentrated, and scarcely perceptible—great sensibility to pressure at the lower part of the sternum—violent abdominal pains—coldness of the lower extremities—faltering of speech. *Antiphlogistics and sedatives, without effect.* 23rd, The symptoms still more intense—pulse scarcely perceptible—agitation excessive—dyspnœa distressing—constant complaint of pain in the region of the heart, with tendency to syncope—great alteration of the features—loss of voice. *Blisters to the legs—sinapisms to the feet—three grains of extract of opium.* These means were productive of no benefit, and the patient expired at eleven o'clock of the same night. "The astonishingly rapid progress of the phenomena left us no time to establish the positive diagnosis of a disease whose sudden invasion and rapid march had veiled the precise seat of the affection."

*Autopsia.* The pleura pulmonalis adhered, by numerous bands, to the pleura costalis. The lungs, when cut and pressed, discharged scarcely any red blood, but only a frothy liquid. The pericardium contained half a pint of a slightly turbid fluid, shewing some albuminous flocculi. The interior surface of the pericardium was phlogosed. The heart was perfectly sound, as was the peritoneum. The mucous membrane of the small intestine was injected, but that of the stomach was pale. There was no other organic lesion.

It is evident the autopsic appearances here, are not in a usual ratio or proportion to the previous symptoms and sudden death. They can only be reconciled by the great susceptibility of the patient after accouchment, in consequence of which, her vital power of resistance was inferior in degree to the disease, and death was the result. Individual idiosyncrasy was here strongly evinced. Many hundreds would bear a much greater advance of organic lesions, without one half the disturbance of functions exhibited by this patient. Most of our readers will agree with the propriety of the following remark of M. Tacheron's, though few of them will be able to recognize the "celebrated practitioner of London, MASTER Bailly." "As in this case the remedies ought to be powerful in proportion as the disease was formidable, we should have practised (in imitation of the celebrated practitioner of London, MASTER BAILLY) three large bleedings at the very beginning;—and, instead of giving opium, we should have applied a very large blister over the region of the heart."*

*Case* 2. (From M. Giponton). A printer entered the hospital on the 1st Pluviose, year 7 of the Republic,† having been ill five days, but

* We do not think our author here alludes to the late lamented physician, Dr Baillie; but to some author of a case in one of the periodical Journals.

† We wish our French brethren would give the common dates, instead of the fantastical terms invented during the mania of the revolution.

previously in good health. On examination, he presented the following symptoms;—viz. headache—some degree of facies hippocratica—circumscribed red spot on each cheek, more especially the left—tongue charged—some cough—abundant expectoration, slightly tinged with blood—respiration much embarrassed—sharp pain in the side (point de coté) extending from the left across the lower portion of sternum to the right, and increased by pressure—obscure sound, on percussion, over a considerable space of the left side, almost the whole of the right side sounding well—constipation—scanty urine, of a turbid appearance, with red sediment—skin dry and burning hot—pulse feeble, small, quick, irregular, and intermittent. *Venesection, diluents.* Two days passed without any remarkable changes, the countenance becoming daily more hippocratic. In the evenings there were exacerbations of the symptoms. 5th. There was nausea added to the other phenomena. *An emetic prescribed,* not without dreading the consequences. It was determined to follow up this measure with *tonics.* The emetic did no good—the patient was entirely deprived of sleep, being obliged to sit constantly upright in bed. In this deplorable condition, he lingered out some ten days more, and expired on the 23rd day, from the invasion of the disease.

*Autopsia.* The brain sound. On cutting through the ribs of the left side, a quantity of purulent matter escaped, evidently flowing from the cavity of the pericardium, which had been opened in the section of the ribs. The pericardium was of very large size, and was computed to contain about a pint and a half of the purulent fluid. Its internal surface was coated with a thick layer of albuminous and puriform matter, with red spots interspersed. The heart was not enlarged, but its pericardial covering was thickened to the extent of two lines—a very unusual circumstance! The muscular structure of the organ did not appear to have undergone any change. The lung, on the left side, though much displaced by the enlarged pericardium, was otherwise sound. The lung, on the right side, was hepatized posteriorly, and covered with a lymphatic exudation. The interior coat of the stomach was inflamed and thickened throughout its whole extent, especially towards the pyloric orifice, which had the appearance of scirrhus. Externally, the stomach appeared quite sound.

*Remarks.* It is probable, as M. Tacheron observes, that the period of acute pericardial inflammation had passed before this man's entrance into hospital—and that purulent secretion or effusion had commenced, when, of course, the disease was incurable. The phenomena of the complaint, however, were strongly characteristic of its nature and seat—consequently, the case is a valuable document, and worthy of record.

*Case* 3. (Related by M. Pied.) Charles Lorrain, 43 years of age, of good constitution, and always having enjoyed excellent health, was seized, on the 20th of May, with difficulty of breathing—shivering—

cough, with little expectoration—pain extending from the epigastrium to the right hypochondrium—fever, with exacerbation at night. Received into hospital on the 22nd, he presented the following symptoms, viz. face of a yellowish red colour—features contracted—slight convulsive movements of the facial muscles—moist tongue, white in the middle—respiration very much impeded, laborious, and quick—slight cough—trifling expectoration, of no particular character—no stool for two days—epigastrium and right hypochondrium painful on pressure—slight heat of skin—pulse small, contracted, quick, regular. *Two bleedings from the arm—diluents—calmants.* The pain in the parts above-mentioned, was mitigated by the bleedings, and a blister removed it entirely; but the other symptoms were not relieved. The night between the 23rd and 24th was passed in a state of great agitation and delirium—and at 4 o'clock in the morning he expired.

*Autopsia.* The left lung was sound, except that its inferior surface was covered with a recently-formed crust of coagulable lymph. The inferior lobe of the right lung was similarly circumstanced, and, in addition, was engorged, as after severe peripneumony. The whole of the thoracic surface of the diaphragm, and the posterior portion of pleura were covered with an inflammatory crust, rather thin, and easily elevated, leaving the subjacent tissue reddish and injected. The abdominal surface of the diaphragm was perfectly sound. The exterior surface of the pericardium was also covered with coagulable lymph, to a considerable extent, and the structure underneath very finely injected. The pericardium itself was filled with a serous or whey-like fluid; and some of the same kind of fluid was extravasated in the cavity of the chest. The internal surface of the pericardium, (both its loose and reflected portions) was covered with an inflammatory crust. The muscular structure of the heart was sound.

It is evident, that here we had an exquisite case of pericarditis, and yet some of the characteristic symptoms of the disease, as laid down by authors, were wanting, viz. syncope, irregularity of pulse, burning pain in the region of the heart, &c. On the contrary, the pulse was regular—there was no very violent pain in the region of the heart—and syncope did not exist.

*Case* 4. Lefebure, 64 years of age, a voiturier, of apparently good constitution, and never before having experienced any serious illness, was upset in his voiture, and one of the wheels passed over the lumbar region. The next day, he had considerable dyspnœa—frequent cough, with bloody expectoration. This state continued, with little alteration, for two months, at which time the symptoms assumed a greater degree of intensity, and forced the patient to enter the hospital, on the 6th of April, when he presented the following phenomena:—features little altered—lips injected—voice hoarse—cough—sanguinolent sputa—sound of the chest, on percussion, clear at the upper, but obscure at the lower part, especially in the region of the heart—action of the heart very irregular—when the hand was applied over the region of the heart, it conveyed the idea of a fluid there—occasional sense of suffocation that greatly interrupted the patient's sleep. *30th April.* The feet and legs infiltrated. Aperient medicines excited the cough, and renewed the sanguineous expectoration.

Between the 1st and 14th May, the disease made great progress. The effusions extended to the hypogastrium—pulse became extinct at the wrists—pain great and deep under the left breast—motions of the heart small and irregular—diarrhœa. Died on the 14th of May.

*Autopsia.* On opening the chest, there appeared traces of recent inflammation on the pericardium, in the form of an albuminous membrane. The origin of the pulmonary artery was dilated to a great extent—and the ventricle itself much enlarged. The inferior portion of each lung was hepatized. All the other organs of the body were sound.

*Case* 5. Charles Baubey, 39 years of age, of apparently good constitution, experienced, about two years ago, after a severe cold, a rheumatic affection of various parts of the body, which left him unable to work at his trade. Afterwards the feet swelled at times, and he had occasional palpitations of the heart. For three months the disease became greatly aggravated, and during fifteen days he could not lie down in bed, so great was the sense of suffocation. The lower extremities became excessively œdematous—his face puffy. On the 3rd of January, 1810, he entered the hospital, presenting the following phenomena: decubitus impossible on the left side—cephalalgia—sleep much interrupted—startings from sleep—confusion in his head—face puffy—tongue white, but moist—respiration greatly impeded—frequent cough—bad sound, on percussion of the chest—great action of the heart—pulse small and frequent—little appetite—thirst intense—alvine dejections scanty, as also the urine—œdema of the lower extremities. *Ptisans, &c.* From the third till the 21st of January, the symptoms became gradually aggravated, and death took place at the last-mentioned period.

*Autopsia.* The right lung was adherent to the costal pleura by false membranes—and the left adhered still more intimately, and was gorged with sanguineous mucosities. The pericardium adhered to the heart throughout, and was thickened in structure, red in colour, and covered with an inflammatory crust. The substance of the heart was flaccid, and easily torn—the parietes of the left ventricle were thickened. Some effusion was found in the cavity of the abdomen; but no other lesion in any part.

*Case* 6. Louis Martigny, 33 years of age, of bilio-sanguine temperament, began to lose his appetite, in the beginning of April, having a bad taste in his mouth. To these symptoms succeeded cough, with difficult respiration, oppression about the chest—decubitus facilis—short and interrupted sleep, in consequence of the cough. During the succeeding days, the symptoms became exasperated with the exception of the expectoration, which was more easy and copious. Towards the end of the month, the difficulty of breathing, and palpitation of the heart were very considerable. 1*st May. Leeches to the anus.* No relief. The legs begin to swell—the sleep is more and more interrupted. 13th. Emaciation has commenced—the face has an earthen hue—lips blue—tongue charged—anterior part of the thorax sounds well, with the exception of the præcordial region, which emits a dull sound on percussion. The inferior part of the left side of the chest emitted no sound at all. The action of the heart was strong and tumultuous—cough very frequent—respiration slow—great tension and swelling in the region of the stomach—urine copious and clear—pulse scarcely perceptible in the left arm—quick, small, and contracted in the right—legs and thighs œdematous—can only lie down on the left side, and that for a very short time. From the 14th to the 30th, sometimes better, sometimes worse. From that time to the 30th June, the symptoms gradually augmented, and death took place at the last-mentioned date.

*Autopsia.* Infiltration general, but more particularly so in the face and extremities. The lungs adhered by bands to the costal pleura—about a pint of yellowish fluid in each cavity of the chest—pericardium adherent to the left lung—anteriorly it was thickened, rough, and covered with a kind of organized membrane internally, which was connected with the heart by several bands of old formation. Between the heart and pericardium there was a considerable quantity of serous effusion. The heart itself was double its natural size. The parietes of the right ventricle were extenuated. There was nothing particular in any of the other organs.

*Case* 7. Claudine Novere, aged 47, had enjoyed good health till the age of 46, when the menstrual secretion ceased. In consequence of a fright, she had an attack of apoplexy, which was not followed, however, with any bad effects. On the 14th of March, after exposure to a hot sun, she fell down senseless, and, four days afterwards, was carried to the hospital, where she presented the following symptoms, viz. hearing perfect, but her utterance was unintelligible, paralysis of the left members of the body, face puffed, eyelids closed, pupils dilated, mouth drawn, tongue black but not dry, pulse full, hard, and quick; skin warm and moist, abdomen tense, belly constipated, urine natural. *Decoction of bark—whey—ptisans—camphor, &c.* 23rd March. Faintings—articulation more free—tongue less black—other symptoms the same. 24th. Subsultus tendinum. 26th, died.

*Dissection.* The vessels of the brain were rather turgid—some spoonfuls of clear serous effusion in the ventricles, and at the base of the brain. Both lungs infiltrated, and but little crepitous. At least a pint of thick yellowish fluid in the pericardium, with albuminous flocculi floating in it. The whole of the pericardium (both the free and reflected portions) was covered with a crust of false membrane, half a line in thickness, and of a yellow colour. There was a livid spot near the apex of the heart, an inch square. The parietes of the ventricles were double their natural thickness, their cavities being extremely contracted, as were both the auriculo-ventricular orifices. Abdominal viscera were sound.

*Remarks.* The above is a very singular case. We see the cardiac disease, which was of enormous extent, almost entirely masked by an affection of the brain, which yet left very faint traces of its existence after death. Very few would have given a correct diagnosis in this instance.

We think the foregoing cases sufficient to make the young practitioner acquainted with the prominent pathological features of pericarditis and hydro-pericarditis. As such, we record them for his perusal.

II. PERITONITIS. The great attention which has been paid, in this country, to puerperal fever, or puerperal peritonitis, has rendered us better acquainted with the pathology of inflammation of the peritoneum, than with almost any other disease. Our medical literature is defective, however, in *post mortem* examinations of peritonitis happening in other than puerperal patients. On this account, we may probably be pardoned for presenting one or two specimens from the stores of our continental brethren, who have such ample opportunities of cultivating pathology in their public hospitals, and who, to do them justice, investigate, with great assiduity, this important branch of medical science.

*Case* 1. A man of athletic form, and 42 years of age, entered the Hotel Dieu on the 27th of May, at 2 o'clock, p. m. The only particulars that could be learnt, were the following, viz. after violent exercise he was suddenly exposed to cold. This was followed by slight pains in the abdomen, and some bilious symptoms. *An emetic had then been taken.* Afterwards came on vomitings, and pains in the lower belly. On entering the hospital, these pains were extreme, and greatly exasperated by the slightest pressure—abdomen tense and distended, presenting an oblong swelling, corresponding with the convolutions of the small intestines—suppression of urine for 18 hours past—breathing short and embarrassed—tongue red and moist—pulse slow and small—face pale—countenance depressed—general perspiration of a thick and viscid kind—heat natural—anxiety—spasms—decubitus dorsalis—inability to lie on either side—wandering of the intellect—*Twenty-four leeches to the abdomen*—in one hour afterwards, 30 *more leeches were applied to the vicinity of the anus—a large bleeding from the arm—injections, &c.* After the venesection, fainting succeeded, with vomiting of greenish matters—the urinary secretion re-appeared. *Re-application of* 30 *leeches.* Died in the evening.

*Dissection.* Abdomen tense and distended—an effusion therein of about two pints of yellow serum mixed with flocculi—all the intestines were coated with an albuminous crust, in the form of false membranes. The lungs were sound—the heart, on its anterior surface, was covered with a kind of false membrane—the parietes of the left ventricle were double their natural thickness, and the cavity exceedingly small. No other organic change in the body,

*Remarks.* M. Tacheron observes that, in this case, the peritonitis was of the most intense kind—and in this we agree with him. We grant, also, that the patient died the same evening—but not "*malgré le traitement rationnel le mieux dirigé à l'hospice*," for every man at all conversant with the bedside of sickness, ought to have known that the patient was dying when he entered the hospital, and that the complaint had advanced to a stage which utterly proscribed the lancet. The effusion—the false membranes, were all in existence on the morning when he entered the hospital, and, therefore, the outrageous system of depletion pursued, was as injudicious as the timid ptisan practice so generally emyloyed by M. Tacheron's countrymen, in the beginning of inflammations, when alone the vigorous system of depletion is advantageous. But there is a tact—a discrimination *in the application* of therapeutical agents, almost independent of a knowledge of pathology or therapeutics themselves. This tact was not well evinced in the treatment of the case just stated.

*Case* 2. Eleanor Arnautt, 21 years of age, was seized, on the 25th of March, with sharp pains throughout the whole of her abdomen, with difficulty of breathing, and restlessness. On the 28th, she entered the Hotel Dieu, presenting the following symptoms—face shrunk, and countenance depressed—decubitus dorsalis—sharp superficial and diffused pain over the whole abdomen, which was very tender to the touch—breathing short, and performed apparently by the dilatation and contraction of the chest alone—sense of fluctuation in the abdomen—debility—pulse small, sharp, and quick—heat moderate—skin dry—constipation obstinate—*twelve leeches to the vagina—whey—fomentations—soups*. 29*th*, temporary alleviation of the symptoms. In the evening an exacerbation. 30*th*, same situation—belly more tense—fluctuation evident. *Ten leeches to the anus*. 31*st*. No better. The pulse is small, contracted, and depressed. *Venesection—emollient lavements*. 1st April. The sufferings a little mitigated—pulse less quick, but harder—nausea and hiccup at times—fetid eructations—abdomen more bulky, and uniformly tense, with great sensibility on pressure—great evening exacerbation. 2*nd*. Pulse small and concentrated—urine yellow—abdomen hard and painful, with a slight ecchymosis at the lower part—all the bad symptoms aggravated. This state continued till the 5th of April, when death put an end to her sufferings.

*Dissection*. The lungs were sound, as was the heart. On opening the abdomen, between five and six pints of an opaque white liquor flowed out, of extremely fetid odour, and containing albuminous flocculi. The whole abdominal cavity was lined with a false membrane, easily separable from the subjacent parts, which were red and thickened. All the viscera were glued together by this inflammatory exudation, which was soft, and evidently of very recent formation. The peritoneal sheet had also a dark appearance.

*Remarks*. The above is an exquisitely marked case of peritoneal inflammation proceeding to a fatal termination, unchecked by remedial means;—for we cannot give the remedies employed here the least credit for even mitigating the dreadful ravages which were going on internally. Our author brings forward several cases of puerperal peritonitis, but they do not differ materially from those already adduced. In this country, the effusion into the cavity of the abdomen is generally seen in fatal cases; but the exudations of coagulable lymph are comparatively rare, in consequence of the more active treatment on this, than on the other side of the channel.

